# Risk factors and outcomes of postoperative stroke in surgical treatment for giant intracranial aneurysms

**DOI:** 10.1186/s41016-022-00297-x

**Published:** 2022-10-03

**Authors:** Hao Wang, Junlin Lu, Xin Chen, Qiang Hao

**Affiliations:** 1 Department of Neurosurgery, Fengtai District, Beijing Tiantan Hospital, Capital Medical University, No. 119 South Fourth Ring Rd West, Beijing, 100070 China; 2 Department of Neurosurgery, West China Hospital, Sichuan University, Chengdu, Sichuan China

**Keywords:** Giant intracranial aneurysms, Microsurgery, Outcome, Stroke, Complication

## Abstract

**Background:**

Giant intracranial aneurysms (GIAs) are challenges for surgical treatment. Risk factors of postoperative stroke remain unclear. This study aims to investigate the predictors of postoperative stroke in GIAs and the impact of stroke on outcomes.

**Methods:**

We performed a retrospective medical record review of patients with GIAs who received microsurgery at our institution between 2011 and 2018. Multivariate logistic regression analyses were carried out to identify risk factors for postoperative stroke. The clinical and angiographic outcomes were compared between patients with and without stroke.

**Results:**

A total of 97 patients were included in this study. Surgical modalities included direct aneurysm neck clipping in 85 patients (87.7%), trapping with the bypass in 8 (8.2%), proximal artery ligation in 1 (1%), and bypass alone in 3 (3.1%). Postoperative stroke was found in 26 patients (26.8%). Independent factors that affect postoperative stroke were recurrent aneurysm (OR, 10.982; 95% CI, 1.976–61.045; *P* = 0.006) and size ≥ 3.5 cm (OR, 3.420; 95% CI, 1.133–10.327; *P* = 0.029). Combined perioperative mortality and morbidity was 26.8%. Follow-up was achieved from 89 patients (91.8%), with a mean follow-up period of 39 months (range 19 to 94 months). Good outcomes were observed in 75 patients (84.3%) and poor outcomes were observed in 14 patients (15.7%).

**Conclusions:**

Postoperative stroke was significantly associated with clinical outcome. Favorable outcomes can be achieved in most patients with GIAs after appropriate microsurgical modality. Recurrent aneurysm and size ≥ 3.5 cm are risk factors of postoperative stroke.

**Supplementary Information:**

The online version contains supplementary material available at 10.1186/s41016-022-00297-x.

## Background

Giant intracranial aneurysms (GIAs) (diameter ≥ 2.5 cm) have always been and remain among the most difficult cerebrovascular lesions to treat, accounting for approximately 5% of all intracranial aneurysms [1]. Previous studies have shown that GIAs warrant treatment because of their poor prognosis if left untreated [2, 3]. Microsurgery as well as endovascular treatment of GIAs remains a challenging procedure because of their irregular shapes and wide necks. Endovascular therapy has gradually become the first choice for these aneurysms, especially since the introduction of the singularly flow diversion into clinical practice [4, 5]. However, considering the higher rate of recanalization or residual compared with small aneurysms and the expensive cost of endovascular treatment. Microsurgery is a definite and reasonable treatment in selected cases. Combined surgical morbidity and mortality have remained in the 20–30% range for many years [6]. Postoperative stroke is one of the most frequent complications after surgical procedures. It plays a significant role in morbidity and mortality [7, 8]. Accordingly, preventing and managing this immediate postoperative complication are essential for ensuring the benefits of surgical treatment. Reports on postoperative stroke in GIAs after microsurgical treatment are scant. Risk factors of postoperative stroke remain unclear. The impact of postoperative stroke on outcomes after surgical treatment remains obscure. Thus, we conducted this study with the following objectives: to explore the risk factors associated with postoperative stroke in GIA patients treated with open microsurgery and to clarify the relation of postoperative stroke with surgical outcomes.

## Methods

### Patients and materials

We performed a retrospective analysis of patients who were diagnosed with intracranial aneurysms at our institution between August 2011 to June 2018. Operative reports, inpatient charts, angiographic studies, magnetic resonance imaging, computed tomographic imaging, and outpatient clinical data were analyzed retrospectively. Smokers as defined as patients who have smoked continuously or accumulatively for 6 months or more in their lifetime. All patients underwent computed tomography angiography (CTA) on admission. Digital subtraction angiography (DSA) was conducted before surgery. Giant aneurysms were defined as having a diameter ≥ 25 mm. With thrombotic aneurysms whose intraluminal diameter on DSA was less than overall aneurysm diameter, axial CT, and/or magnetic resonance imaging (MRI) were used to measure the aneurysm size. Recurrent aneurysms were defined as which had been previously operated upon or had been previously coiled before this admission.

The inclusion criterion for participation in this study was the diagnosis of an intracranial aneurysm with a diameter of at least 25 mm using DSA, CTA, or MRI, independent of aneurysm shape. Patients with GIAs who received endovascular or conservative treatment were excluded from this review. The primary endpoint was the occurrence of postoperative stroke within 30 days after surgery. Postoperative cerebral stroke was defined as a symptomatic event within 30 days after surgery and confirmed by CT or MRI. Symptoms included focal neurological deficits lasting more than 24 h. Neurological outcomes were assessed using the modified Rankin Scale (mRS) score. The mRS scores were recorded on the day of admission, discharge, and follow-up. All scale assessments were performed by neurosurgeons who were not directly engaged in the care of these patients.

### Surgical modalities

An individual surgical plan was made for every patient. Giant aneurysms were exposed using standard site-appropriate surgical approaches. The selection of different surgical modalities depended on the preoperative radiological imaging and intraoperative findings. Direct aneurysm neck clipping with suitable clips was the primary treatment strategy, sometimes requiring temporary trapping, thrombectomy, and clip reconstruction. Indirect aneurysm occlusion was invoked as the alternative treatment strategy when direct neck clipping was not possible or considered too risky. Indirect aneurysm occlusion was as follows: (1) trapping the aneurysm by distal and proximal occlusion when good collateral blood supply existed; for those without enough collaterals, combined revascularization was also considered; (2) proximal artery (the cervical ICA) ligation if the Matas test or balloon occlusion test can be tolerated; if not, combined bypass surgery was considered. (3) Bypass surgery alone. Intraoperative somatosensory evoked potential and motor evoked potential monitoring were routinely applied. Adequacy of treatment and patency of parent vessels was analyzed intraoperatively using intraoperative angiography, and/or indocyanine green (ICG) fluorescence video angiography. The postoperative body’s fluid balance and blood pressure were strictly controlled to prevent vasospasm.

### Follow-up protocols

Patients were followed with DSA or CTA or MRI and clinical examinations 3 ~ 6 months following initial treatment and annually thereafter. Doctors performing follow-up assessments were blinded to baseline information. Angiographic outcomes including aneurysm residual, aneurysm recurrence, de novo aneurysm, and recurrent hemorrhage were collected during follow-ups. Preoperative neurologic condition was used as a reference point, and patient outcomes were expressed in terms of changes from this baseline (improved, unchanged, worse, or dead). Thus, asymptomatic patients who remain without neurologic deficits were classified as unchanged.

### Statistical analysis

Data was analyzed using IBM SPSS Statistics version 26.0 (IBM Corp.). Statistical significance was set at *p* < 0.05. The differences of the original baseline and outcomes between the stroke and no-stroke groups were evaluated by using Mann–Whitney *U* test for continuous variables, a chi-square test for categorical variables. Odds ratios (ORs) and 95% confidence intervals (CIs) for postoperative stroke for potential risk factors were calculated by univariate and multivariate logistic regression analyses. To further determine the potential risk factors of postoperative stroke in GIAs patients with different treatment modalities, we conducted subgroup analysis in the patients with direct clipping.

### Data availability disclosure

The data that support the findings of this study are available from the corresponding author upon reasonable request.

## Results

### Patients baseline

**Fig. 1:**
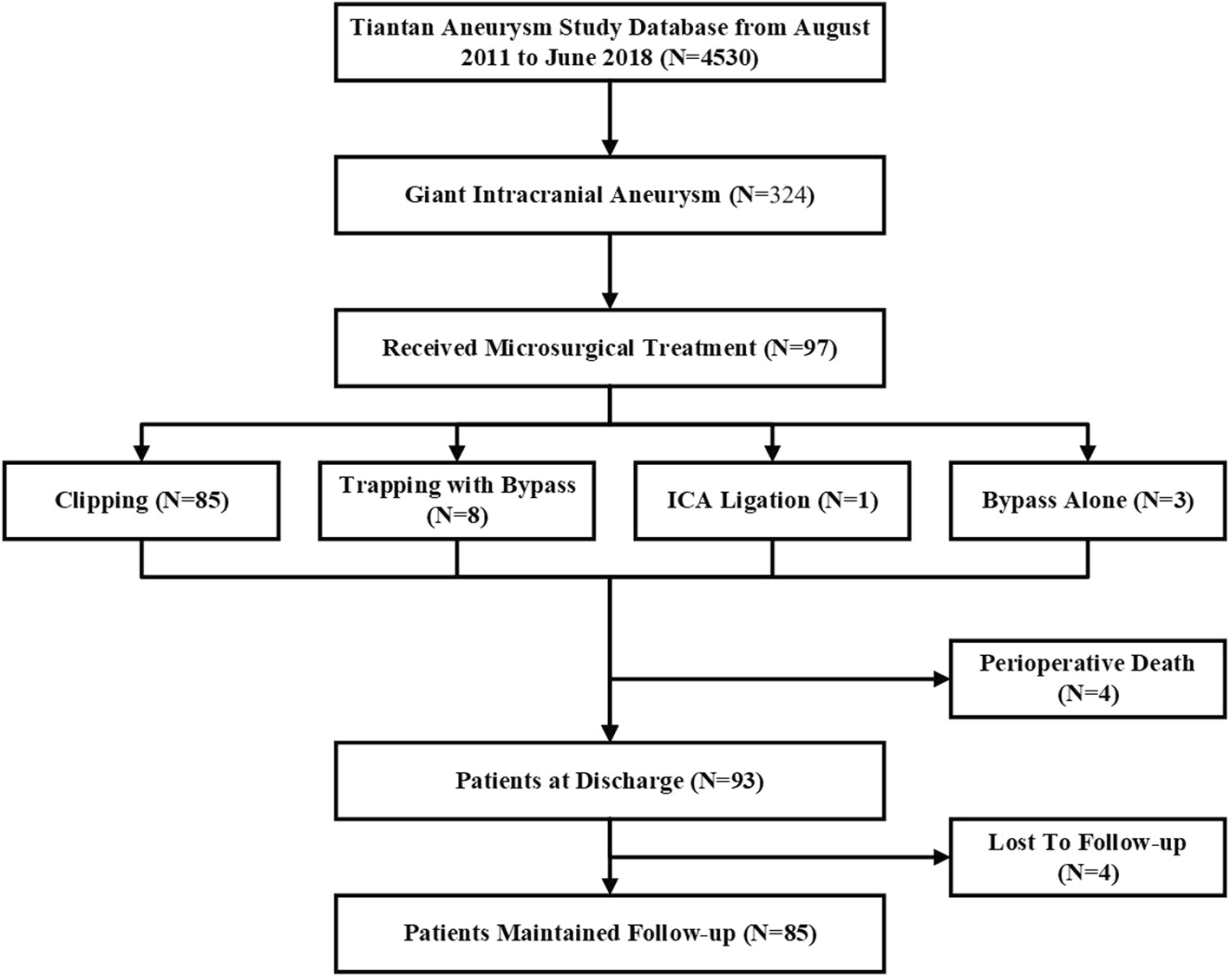
Flow diagram of the study population

During an 8-year period from August 2011 to June 2018, 4530 patients were diagnosed with aneurysms and treated in our institution. Of these patients, 97 patients with GIAs were treated microsurgically (2.1% of all patients) (shown in Fig. 1). Baseline characteristics of patients with and without postoperative stroke were presented in Table 1. There were 64 women (66%) and 33 men (34%), with a mean age of 48 years (range, 8–71 years), and 93.8% were adult patients. The female-to-male ratio was 1.9:1. Neurological deficits and headache were the most frequent clinical manifestation, accounting for 37.1% and 28.9%, respectively. Eleven patients presented with subarachnoid hemorrhage (11.3%), 10 (10.3%) patients had dizziness only, and the rest 12 (12.4%) had no obvious symptoms. Among 36 patients presented with varying degrees of neurological deficits, visual deficits were the most common symptoms (23/36, 63.9%). Six patients presented with recurrent aneurysms after previous endovascular coiling, and 2 patients presented with recurrent aneurysms after previous microsurgical clipping.

**Table 1:** Baseline characteristics of patients with and without postoperative stroke

Characteristic	All Pts (*n* = 97)	Postop stroke		*p* value
		Present (*n* = 26)	Absent (*n* = 71)	
Mean age, years	47.79 ± 14.49	45.85 ± 16.00	48.51 ± 13.95	0.426
Age < 18 years	6 (6.2)	2 (7.7)	4 (5.6)	0.709
Sex				0.940
Male	33 (34.0)	9 (34.6)	24 (33.8)	
Female	64 (66.0)	17 (65.4)	47 (66.2)	
Onset symptoms				0.066
SAH	11 (11.3)	3 (11.5)	8 (11.3)	
Neurological deficits	36 (37.1)	7 (26.9)	29 (40.8)	
Headache	28 (28.9)	13 (50.0)	15 (21.1)	
Dizziness	10 (10.3)	2 (7.7)	8 (11.3)	
Asymptomatic	12 (12.4)	1 (3.8)	11 (15.5)	
Medical history				
Smoking	14 (14.4)	5 (19.2)	9 (12.7)	0.416
Drinking	10 (10.3)	2 (7.7)	8 (11.3)	0.608
Diabetes	4 (4.1)	0 (0)	4 (5.6)	0.216
Hypertension	41 (42.3)	11 (42.3)	30 (42.3)	0.996
Hyperlipidemia	3 (3.1)	0 (0)	3 (4.2)	0.287
Preop mRS score				0.934
< 2	93 (95.9)	25 (96.2)	68 (95.8)	
≥ 2	4 (4.1)	1 (3.8)	3 (4.2)	
Recurrent aneurysm	8 (8.2)	6 (23.1)	2 (2.8)	0.001
Multiple aneurysm	18 (18.6)	3 (11.5)	15 (21.1)	0.282
Size, cm	2.96 ± 6.23	3.12 ± 6.46	2.89 ± 6.07	0.107
< 3.5 cm	78 (80.4)	17 (65.4)	61 (85.9)	0.024
≥ 3.5 cm	19 (19.6)	9 (34.6)	10 (14.1)	
Location				0.913
Anterior circulation	90 (92.8)	24 (92.3)	66 (93.0)	
Posterior circulation	7 (7.2)	2 (7.7)	5 (7.0)	
Shape				0.416
Saccular	91 (93.8)	23 (88.5)	68 (95.8)	
Fusiform	2 (2.1)	1 (3.8)	1 (1.4)	
Serpentine	4 (4.1)	2 (7.7)	2 (2.8)	
Surgical modalities				0.077
Clipping	85 (87.7)	21 (80.8)	64 (90.1)	
Trapping with bypass	8 (8.2)	5 (19.2)	3 (4.2)	
ICA ligation	1 (1.0)	0 (0)	1 (1.4)	
Bypass alone	3 (3.1)	0 (0)	3 (4.2)	
LOS, days	19.39 ± 8.90	23.04 ± 8.39	18.06 ± 8.76	0.014

Data are *n* (%) unless otherwise indicated. Mean values are given with SDs

*SAH* Subarachnoid hemorrhage, *Preop* Preoperative, *mRS* Modified ranking scale, *LOS* Length of hospital stays

Eighteen patients (18.6%) harbored multiple aneurysms, 45 in total. None of these patients had two giant aneurysms. The size of the aneurysms ranged from 25 to 55 mm (median 30 mm). The anatomical distribution of the 97 GIAs was shown in Table 2. Ninety aneurysms (92.8%) were located in the anterior circulation, the most common sites being the middle cerebral artery (MCA, 43.4%), followed by internal carotid artery (ICA) bifurcation (19.6%), and ophthalmic ICA (9.3%). Seven aneurysms were located in the posterior circulation, the most common sites being the posterior cerebral artery (PCA, 4.1%) and the vertebral artery (VA, 2.1%). According to CT angiography and three-dimensional DSA images, 91 (93.8%) aneurysms were saccular, 2 (2.1%) were fusiform, and 4 (4.1%) were serpentine.

**Table 2:** Anatomic distribution of the giant aneurysms

Location	Total (*n* = 97)	Postop stroke	
		Present (*n* = 26)	Absent (*n* = 71)
Anterior circulation	90 (92.8)	24 (92.4)	66 (93.0)
ICA	39 (40.2)	10 (38.0)	29 (40.8)
Cavernous segment	4 (4.1)	1 (3.8)	3 (4.2)
Clinoid segment	7 (7.2)	3 (11.5)	4 (5.6)
Ophthalmic segment	9 (9.3)	0 (0)	9 (12.7)
Bifurcation	19 (19.6)	6 (23.1)	13 (18.3)
PCoA	4 (4.1)	2 (7.6)	2 (2.8)
ACA	1 (1.0)	0 (0)	1 (1.4)
ACoA	4 (4.1)	0 (0)	4 (5.6)
MCA	42 (43.4)	12 (46.4)	30 (42.0)
Posterior circulation	7 (7.2)	2 (7.6)	5 (7.0)
VA	2 (2.1)	1 (3.8)	1 (1.4)
PICA	1 (1.0)	0 (0)	1 (1.4)
PCA	4 (4.1)	1 (3.8)	3 (4.2)

Data are *n* (%) unless otherwise indicated

*ICA* Internal carotid artery, *PCoA* Posterior communicating artery, *ACA* Anterior cerebral artery, *ACoA* Anterior communicating artery, *MCA* Middle cerebral artery, *VA* Vertebral artery, *PICA* Posterior inferior cerebellar artery, *PCA* Posterior cerebral artery

### Surgical modalities

Overall, 97 GIAs were treated in 97 patients. Most of the patients were treated directly with neck clipping (85/97, 87.7%). Aneurysm trapping combined with bypass was conducted in 8 patients (8.2%). Thrombectomy was performed with 36 aneurysms (37.1%) to facilitate direct clipping or to decompress the brain or cranial nerves after direct clipping. Only one patient (1.0%) was treated with ICA ligation. Bypass alone was performed in 3 patients (3.1%) because of the high risk of clipping and trapping.

### Postoperative stroke

Postoperative stroke occurred in 26 patients (26.8%). In 21 cases, the complications occurred after clipping; and in 5 cases, after trapping with bypass. Univariable and multivariable ORs for the risk factors of postoperative stroke were given in Table 3. Univariate analysis showed that recurrent aneurysm (OR, 10.350; 95% CI, 1.937–55.308; *P* = 0.006), and size greater than 3.5 cm (OR, 3.229; 95% CI, 1.131–9.217; *P* = 0.028) were associated with postoperative stroke. After adjustment for confounding variables in multivariate analysis, recurrent aneurysm (OR, 10.982; 95% CI, 1.976–61.045; *P* = 0.006) and size greater than 3.5 cm (OR, 3.420; 95% CI, 1.133–10.327; *P* = 0.029) remained associated with a significantly increased risk of stroke. In addition, in the direct clipping subgroup analysis, the recurrent aneurysm (OR, 10.362; 95% CI, 1.785–60.133; *P* = 0.009) was also significantly associated with postoperative stroke (Table Supplementary).

**Table 3:** Logistic regression analysis for postoperative strokes

Covariate	Univariable		Multivariable	
	OR (95% CI)	*p* value	OR (95% CI)	*p* value
Mean age, years	0.988 (0.958–1.018)	0.423		
Sex	1.037 (0.403–2.670)	0.940		
Ruptured aneurysm	0.796 (0.201–3.151)	0.745		
Smoking	1.640 (0.494–5.446)	0.419		
Drinking	0.656 (0.130–3.314)	0.610		
Diabetes	——	0.999		
Hypertension	1.002 (0.404–2.488)	0.996		
Hyperlipidemia	——	0.999		
Recurrent aneurysm	10.350 (1.937–55.308)	0.006	10.982 (1.976–61.045)	0.006
Size ≥ 3.5 cm	3.229 (1.131–9.217)	0.028	3.420 (1.133–10.327)	0.029
Location				
Anterior	Ref	Ref		
Posterior	1.100 (0.200–6.052)	0.913		
Shape				
Non-saccular	Ref	Ref		
Saccular	0.338 (0.064–1.794)	0.203		
Surgical modalities				
Non-clipping	Ref	Ref		
Clipping	0.459 (0.132–1.602)	0.222		

### Outcomes

**Fig. 2:**
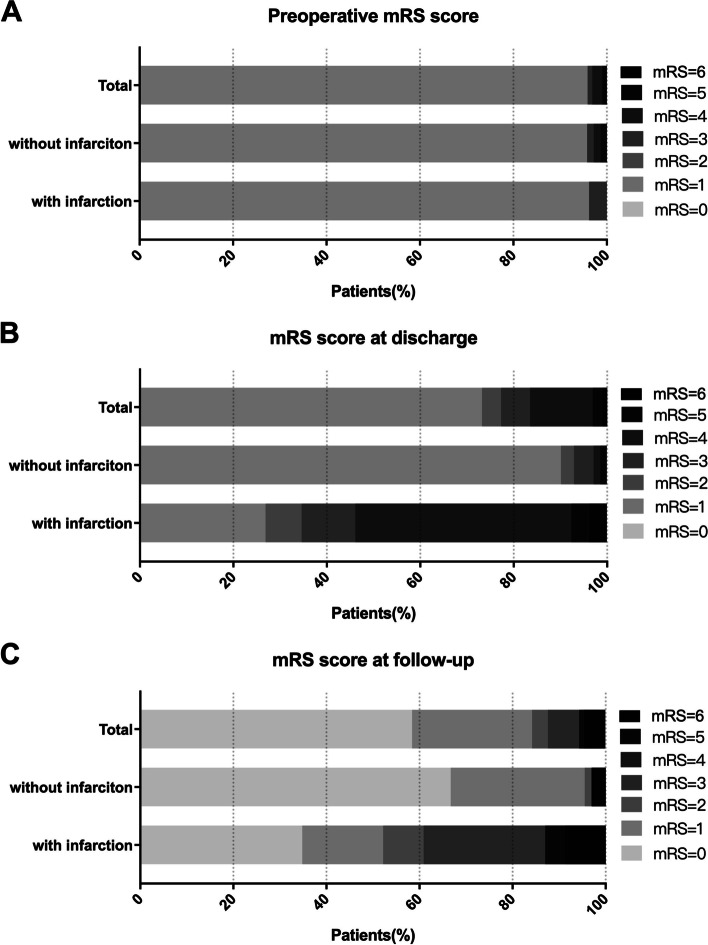
Comparison of mRS scores of patients with and without postoperative stroke. The proportions of patients with mRS scores ranging from 0 to 6 are shown for all patients on admission (**A**), at discharge (**B**), and the last follow-up (**C**)

Table 4 showed the perioperative and follow-up outcomes of GIA patients. According to the intraoperative ICG fluorescence video angiography, 94 of 97 aneurysms (96.9%) were completely occluded with no residual aneurysm or neck remnant. Three aneurysms (3.1%) had minimal residual aneurysm after clipping (small neck remnant or dog-ear) and further treatment was deemed unnecessary. The rate of treatment-related mortality (mRS score 6) and morbidity (mRS score 2–5) was 4.1% (4/97) and 22.7% (22/97) in the perioperative period. During an average of 39 months (range 19 to 94 months) follow-up period, four patients (4.1%) were lost to follow-up. Therefore, follow-up data were achieved from the other 89 patients (91.8%). No recanalization is observed after complete occlusion of aneurysms treated with microsurgery. Three patients (3.4%) experienced intracranial hemorrhage during the follow-up period, creating an annual hemorrhage risk of 1.1%. One patient had an aneurysm recurrent (1.1%, annual recurrence risk 0.3%) 3 years after surgery and underwent another endovascular treatment at our institution. In addition, 3 de novo aneurysm formations (3.4%, annual de novo formation risk 1.1%) were observed in other patients. These de novo aneurysms were neither GIAs or generated from GIAs. Two of them underwent another endovascular treatment at our institution while one of them was treated with conservative observation. Of the 89 patients, 4 patients (4.5%) died (mRS score 6) at the last follow-up, 14 patients (15.7%) had different degrees of neurological deficits (mRS score 2–5), and the other 75 patients (84.3%) were disability-free (mRS score 0–1). Causes of death included massive cerebral stroke in 2 patients and intracranial hemorrhage in 2 patients. Between the 26 patients with postoperative stroke and the 71 patients without postoperative stroke, there was a significant difference in mRS score in the perioperative period, mRS score at the last follow-up, and neurological function deterioration ratio (shown in Fig. 2).

**Table 4:** Perioperative and follow-up outcomes in the postoperative stroke and no-postoperative strokes groups

Periop outcomes	Total	Postop stroke		*p* value
		Present	Absent	
No. of patients	97	26	71	
Aneurysm residual	3 (3.1)	0 (0)	3 (4.2)	0.287
mRS score 1 month postop				< 0.001
< 2	71 (73.2)	7 (26.9)	64 (90.1)	
≥ 2	26 (26.8)	19 (73.1)	7 (9.9)	
Mortality 1 month postop	4 (4.1)	2 (7.7)	2 (2.8)	0.285
Follow-up outcomes				
No. of patients	89	23	66	
Hemorrhage	3 (3.4)	0 (0)	3 (4.5)	0.298
Aneurysm recurrence	1 (1.1)	0 (0)	1 (1.5)	0.553
De novo aneurysm formation	3 (3.4)	2 (8.7)	1 (1.5)	0.100
mRS score at last follow-up				< 0.001
< 2	75 (84.3)	12 (52.2)	63 (95.6)	
≥ 2	14 (15.7)	11 (47.8)	3 (4.5)	
Mortality at last follow-up	4 (4.5)	2 (8.7)	2 (3.0)	0.259
Neural function deterioration	14 (15.7)	11 (47.8)	3 (4.5)	< 0.001

## Discussion

GIAs, defined by a diameter above 25 mm, are often discovered through their mass effect or hemorrhage, which represent about 5% of all intracranial aneurysms [1, 9]. They can occur in all age groups but are most commonly seen between 40 and 70 years with a female predominance [6, 10–12]. Our results were consistent with previous studies. In our series, we detected 324 patients (7.2%) diagnosed with GIAs among all 4530 patients. Of these, 97 patients (2.1%) received microsurgical treatment were included in this study. Similarly, the majority of patients’ ages were from 40 to 70 years (72/97, 74.2%). The female-to-male ratio in patients with GIAs was 1.9:1. As for the location, the anterior circulation, especially the MCA, is the most common region in our cohort, whereas there was a clear predominance on the ICA in other papers [7, 13]. However, it should be noted that endovascular alternatives have changed indications for microsurgical treatment. Endovascular techniques have been utilized with promising early results, particularly with giant aneurysms located on cavernous and clinoid segments of the ICA. Therefore, fewer GIAs located on the ICA come to surgical management. According to our retrospective database, GIAs treated with endovascular treatment were approximately twofold of with open microsurgery. This might also explain some different views from older publications that GIAs were regularly formed in the posterior circulation [14]. Previous studies reported that the most common manifestations of GIAs is neurological deficit caused by mass effect, followed by the subarachnoid or intracerebral hemorrhage [15]. In the present study, 85 of the 97 GIAs were symptomatic, the most common clinical manifestation was neurological deficits (37.1%), followed by headache (28.9%), intracranial hemorrhage (11.3%), and dizziness (10.3%).

Several studies have shown that the prognosis of GIAs was poor if leaving untreated. The rupture risk was exceeding 10% per year [2, 16]. The large mass effect of the unruptured GIAs also contributed to the unfavorable outcome. A recently published meta-analysis, which enrolled 54 studies containing 64 study populations with 1269 GIAs, concluded that there was no difference in the clinical outcome between the two modalities [17]. Endovascular therapy for GIAs has a higher rate of recurrence and residual compared with small aneurysms [18–20]. Although the safety and effectiveness of the pipeline embolization device had been verified in many papers [4, 21]. The cure rate ranging from 55.7 to 84.0% and it takes 6–18 months to reach a complete occlusion [22]. In the present study, we observed 96.9% of the GIAs were completely occluded during the perioperative period and no recanalization was observed during the follow-up. Thus, microsurgical aneurysm occlusion is still a single, definitive, and durable therapy for GIAs.

We chose clipping as the primary option for GIAs, which accounted for 87.7% of our series. Although there were no significant differences in the rate of postoperative stroke among different surgical modalities. Patients who underwent trapping with bypass have a higher incidence of postoperative cerebral stroke than patients who underwent clipping (62.5% versus 24.7%). Of these 8 patients, a superficial temporal artery-to-MCA (STA-MCA) bypass procedure was carried out to avoid ischemia after clipping the parent artery. However, as STA-MCA bypass is a low-flow bypass, the cerebral blood flow might be not sufficient, even if the bypass was conducted in some patients. Therefore, a high-flow bypass such as an external carotid artery-to-MCA bypass should be recommended in these patients. ICA ligation is the last choice and aims to reduce the blood flow to the aneurysm, also to reduce the rupture risk. In our study, one female patient tolerated the Matas test and underwent ICA ligation. She discharged with no neurological deficits. However, 2 years after the surgery she suffered an ischemic stroke and left permanent neurological deficits. Because of the uncertain long-term prognosis, we should be more prudent when considering the ICA ligation. Bypass alone was performed on three patients. Follow-up angiograms obtained 3 months and 6 months later showed unchanged of the aneurysm. None of these aneurysms ruptured during the follow-up. Also, patients claimed the alleviation of the preoperative symptoms. However, the effectiveness of bypass alone in treating GIAs needs further study. Due to the long-time span of the patients enrolled in this study, a Mata’s test was routinely performed in patients before proximal ligation or surgical trapping of the aneurysms in the early period. In recent years, balloon test occlusion was performed to evaluate the cerebral blood flow before proximal ligation or surgical trapping.

Several studies reported that the combined surgical morbidity and mortality of GIAs varies from 20 to 30%. Age of the patient, aneurysm location, and preoperative Hunt-Hess grade was associated with the outcome after surgical treatment [3, 7, 8, 11, 14, 17, 23]. Postoperative stroke is one of the most common complications, which might attribute to aneurysm thrombosis with perforator or branch artery occlusion or bypass occlusion. Cigarette smoking has already been recognized as a risk factor for ischemic and hemorrhagic stroke [24]. Smoking can reduce the biological activity of nitric oxide and inhibit endothelial cell growth, leading to arterial endothelial injury and promotes thrombosis [25]. However, we did not find that smoking was associated with postoperative stroke in the present study. It might because not many smokers were included in this study (stroke group = 5, non-stroke group = 9). Furthermore, we also found that the size of aneurysm greater than 3.5 cm and recurrent aneurysms were independent risk factors of postoperative stroke. At present, scholars believe that the most important reason for intracranial aneurysms may be vascular degenerative injury caused by hypertension or other factors. The injured vascular endothelium can release vascular endothelial factors, increasing the probability of thrombosis [26]. In the recurrent aneurysms, the vascular endothelium might be injured again, leading to a higher risk of stroke. Also, most recurrent aneurysms regenerate from the neck of the aneurysms and closely next to the other perforating branches of the parent artery. The narrow space makes the operation more difficult and increased the risk of stroke. Moreover, aneurysm thrombosis is common in GIAs, the plaque breaks down during the clipping procedure it may be a key factor causing postoperative stroke. In the direct clipping subgroup, only the recurrent aneurysm was associated with postoperative stroke. One of the possibilities is that with the development of surgical techniques and tools, even if it has a relatively giant size, GIAs with a good shape can be clipped without postoperative complications.

Some study limitations need to be addressed for accurate interpretation of our data. This is a single-center study, surgical indications, surgical procedures, and perioperative patient management may vary accordion to institutional philosophy and experience. In this study, only patients treated with microsurgery were included, most of the aneurysms were at anterior circulation and this introduced selection bias. Also, similar to all retrospective studies, during 8 years follow-up and not all patients were followed up regularly. This may lead to the results prone to potential attrition biases.

## Conclusions

In summary, microsurgery is a definite and reasonable treatment in selected cases for GIAs. Favorable outcomes can be achieved in most patients with GIAs after appropriate microsurgical modality. Our data support smoking, recurrent aneurysm, and size of aneurysm ≥ 3.5 cm were risk factors of postoperative stroke. Postoperative stroke was significantly associated with worse outcomes at discharge and follow-up.

## Supplementary Information

**Additional file 1: Table Supplementary.** Logistic regression analysis for postoperative strokes in clipping sub-group.

## Data Availability

The data that support the findings of this study are available from the corresponding author upon reasonable request.
