# Supplementary material for: Risk factors and outcomes of postoperative stroke in surgical treatment for giant intracranial aneurysms
Source: Chin Neurosurg J. 2022 Oct 3;8:31. doi: 10.1186/s41016-022-00297-x (PMC9528132; doi:10.1186/s41016-022-00297-x)
Supplement: Supplementary file 1 — Additional file 1: Table Supplementary. Logistic regression analysis for postoperative strokes in clipping sub-group. [file 41016_2022_297_MOESM1_ESM.docx]

Table Supplementary. Logistic regression analysis for postoperative strokes in clipping sub-group.

|  | Univariable | | Multivariable | |
| --- | --- | --- | --- | --- |
| Covariate | OR (95% CI) | p Value | OR (95% CI) | p Value |
| Mean age, years | 1.018 (0.978-1.059) | 0.384 |  |  |
| Sex | 0.833 (0.295-2.355) | 0.940 |  |  |
| Ruptured aneurysm | 0.900 (0.223-3.636) | 0.882 |  |  |
| Smoking | 1.910 (0.560-6.513) | 0.301 |  |  |
| Drinking | 0.737 (0.144-3.778) | 0.714 |  |  |
| Diabetes | —— | 0.999 |  |  |
| Hypertension | 1.414 (0.526 -3.801) | 0.492 |  |  |
| Hyperlipidemia | —— | 0.999 |  |  |
| Recurrent aneurysm | 9.687 (1.718-54.615) | 0.010 | 10.362 (1.785-60.133) | 0.009 |
| Size ≥ 3.5cm | 3.021 (0.815-11.190) | 0.098 | 3.312 (0.840-13.058) | 0.087 |
| Location |  |  |  |  |
| Anterior | Ref | Ref |  |  |
| Posterior | 1.242 (0.223-6.932) | 0.805 |  |  |
| Shape |  |  |  |  |
| Non-Saccular | Ref | Ref |  |  |
| Saccular | 0.306 (0.040-2.325) | 0.253 |  |  |
